# The Contribution of the Sheep and the Goat Model to the Study of Ovarian Ageing

**DOI:** 10.3390/biology12020270

**Published:** 2023-02-08

**Authors:** Luís Montenegro, Paulo Magalhães, Adriana Costa Guerreiro, Catarina Brandão, Anabela Pinto, Henrique Almeida, Ana Martins-Bessa, Elisabete Silva

**Affiliations:** 1 Animal and Veterinary Research Center (CECAV), Universidade de Trás-os-Montes e Alto Douro (UTAD), 5000-801 Vila Real, Portugal; 2 Veterinary Hospital Referência Veterinária Montenegro, Rua da Póvoa 34, 4000-395 Porto, Portugal; 3 AL4AnimalS-Associate Laboratory for Animal and Veterinary Sciences, 1300-477 Lisboa, Portugal; 4 Unidade de Biologia Experimental, Departamento de Biomedicina, Faculdade de Medicina, Universidade do Porto, Alameda Professor Hernâni Monteiro, 4200-319 Porto, Portugal; 5 Instituto de Biologia Molecular e Celular (IBMC), i3S-Instituto de Investigação e Inovação em Saúde, Universidade do Porto, Rua Alfredo Allen, 4200-135 Porto, Portugal; 6 Ginecologia-Obstetrícia, Hospital CUF, Estrada da Circunvalação 14341, 4100-180 Porto, Portugal; 7 Departamento de Imuno-Fisiologia e Farmacologia, Instituto de Ciências Biomédicas Abel Salazar (ICBAS), Universidade do Porto, Rua de Jorge Viterbo Ferreira, 4050-313 Porto, Portugal

**Keywords:** animal models, ovarian function, multinucleated giant cells, ovarian ageing

## Abstract

**Simple Summary:**

Ovarian ageing is considered the major contributor to the age-related loss of fertility. Previous studies in mice evidenced the presence of a unique population of multinucleated giant cells in the stroma of the ovaries from older animals. Their presence correlated negatively with the ovarian follicle pool. The aims of the present work were to identify and characterize the presence of ovarian multinucleated giant cells in the goat and sheep ovary. The study of the ovarian morphology points to an age-related decrease in the primordial follicle pool, as observed in humans and mice. Additionally, with the aid of Sudan black B staining and autofluorescence detection, multinucleated giant cells were identified in the ovaries of mature females of both species. Implications of this research outcome are discussed, considering the presence of multinucleated giant cells in an age at which a decrease in reproductive potential is expected. Future studies should explore the potential mechanism and possible impairment on the ovarian function and, consequently, fertility.

**Abstract:**

Ovarian ageing stands as the major contributor towards fertility loss. As such, there is an urge for studies addressing the mechanisms that promote ovarian ageing and new strategies aiming to delay it. Recently, the presence of a unique population of multinucleated giant cells has been identified in the ovaries of reproductively aged mice. These cells have been considered hallmarks of ovarian ageing. However, up to date multinucleated giant cells have only been described in the ovaries of the mice. Therefore, the aim of the present work was to evaluate and characterize the presence of such hallmarks of ovarian ageing in the sheep and the goat. In this study, ovaries from juvenile (6 months) and mature animals (18–24 months) were used. The hematoxylin and eosin technique was performed to describe the ovarian morphology and evaluate the ovarian follicle reserve pool. Sudan black B staining and the detection of autofluorescence emission were used to identify and characterize the presence of multinucleated giant cells. Statistical analyses were performed with GraphPad Prism 9.0.0. A decrease in the follicle reserve pool and the presence of multinucleated giant cells, with lipofuscin accumulation and the emission of autofluorescence, were observed in the ovaries of the mature animals of both species. Our results support the interest in the use of the ovine and the caprine model, that share physiological and pathophysiological characteristics with humans, in future studies addressing ovarian ageing.

## 1. Introduction

During the last decades, we have seen an increase in the lifespan of humans and domestic animals. However, this has not been accompanied neither by an increase in females’ reproductive life span nor an equally healthy aging. These facts have a profound economical and medical impact, not only because a major portion of a female’s life is spent in a period of reproductive impairment, which induces the development of diseases and health complications associated with reproductive aging, but also because in developed and developing countries, a variety of economic and educational reasons have led women to the postponement of childbearing into a latter period of their lives [1] where fertility is significantly decreased and the incidence of pregnancy-associated complications is increased [2]. Therefore, actions are needed to mitigate this temporal impairment between lifespan and reproductive life span.

Biological aging is the time-dependent functional decline that primes the development of age-related diseases and increases death susceptibility. In females, the reproductive system is the first to show signs of biological aging, as evidenced by cycle irregularities and fertility decrease [3,4]. Ovary transplant experiments and oocyte donation programs demonstrated that the loss of the ovary function is the main contributor towards reproductive aging. Despite sharing some common features to the aging of other organs and systems, the mechanisms underlying ovarian disfunction have not been fully elucidated.

The ovaries are considered the main factor underlying age-related fertility loss in humans, due to the continuous decay in ovarian follicle quantity and quality [4,5,6]. In addition to that monthly attrition of a large number of follicles, a likely contributor is an age-related loss of redox balance, which promotes oxidative stress and induces damage to biomolecules [7,8] leading to genomic instability, the loss of proteostasis, and the impairment of cell stress response pathways. An ovarian reaction to such damage has been found in follicles and also in the complex interstitial connective tissue [7,9,10,11], whose cells manifest an impaired mitochondrial function [12,13]. This has been shown to culminate in an age-related change in ovarian phenotype, namely impaired folliculogenesis [14], reduced oocyte quality [15], the altered expression of receptors for oocyte-secreted factors [16], and additional ovarian dysfunctional effects [15,17]. In humans, oxidative stress-mediated ovarian damage contributes to the low success rate of assisted reproductive technologies in women in their 40 s [8,15].

Within the assessment of age-related effects on the ovary, the changes occurring in the stroma have attracted growing interest. While studying the ovarian microenvironment, Briley and colleges [18] reported an increase in inflammation and fibrosis and proposed that such changes contribute to the decrease in the oocyte quality. This observation was further supported by demonstrating that an antioxidant administration ameliorated the age-related disruption of the ovarian microenvironment caused by a feedforward loop between oxidative stress, inflammation, and fibrosis [9].

Recently, focus has been put into a unique ovarian population of multinucleated giant cells present in the reproductively aged mice. Multinucleated giant cells accumulate lipofuscin, they contain deposits of nitrated proteins, and they are positive for the F4/80 mice macrophage marker [9,18,19]. Multinucleated giant cells have been considered hallmarks of ovarian ageing in mice as they correlate negatively with the ovarian follicle reserve [20,21]. However, their presence in the ovaries of other mammalian species has not been documented. This point is rather important because should similar changes be noticed, notably in humans, such findings would shed light on the conditions underlying the continued age-related fertility loss.

In that setting, sheep and goats may be useful since they are closer to humans than the mice, the rat, and the rabbit [22]. Therefore, this work aimed to verify the presence of multinucleated giant cells in the ovaries of goats and sheep at distinct ages.

## 2. Materials and Methods

### 2.1. Tissue Collection

The ovaries from juvenile (6 months) and mature (18–24 months) sheep and goat (Table 1), as by-products of animals used for human consumption, were used in the present study. They were collected during the first trimester of 2022 at slaughterhouse PEC Nordeste S.A. The collection was authorized by the Portuguese National Authority for Animal Health (DGAV) and the tissues were processed at the Faculty of Medicine of Porto University. Faculdade de Medicina da Universidade do Porto is authorized to use the by-products of animals used for human consumption (authorization N.13.029.UDER). The present research did not require ethical approval.

The precise age of the small ruminants could not be determined because the animals were transported in groups to the slaughterhouse by their raisers, who provide the approximate birth date of the group.

### 2.2. Paraffin Embedding

A total of 31 ovaries (Table 1) were fixed in 4 % buffered formaldehyde for 24 h and sliced in half according to the longitudinal axis of the ovary. The tissue was then dehydrated with the aid of increasing the concentrations of ethanol (70%—1 passage for 24 h, 70%—2 passages for 30 min, 90%—4 passages for 30 min each, and 100%—4 passages for 30 min each), diaphanized using xylol (1 h), and embedded in paraffin (2 passages for 1 h each). Sequential slides (5 μm thick) of ovarian midsections were obtained with the aid of a microtome (Leica, RM2145, Mannheim, Germany, ). Ovarian tissue was mounted on poly-L-lysine (Sigma-Aldrich, P8920, St. Louis, MO, USA)-coated slides and dried for 48 h at 37 °C. The slides were stored in plastic boxes to be used for all histological applications throughout the study.

### 2.3. Haematoxylin and Eosin Staining

The hematoxylin and eosin staining is the standard histochemical technique used for the microscopic examination of paraffin-embedded tissues. Ovarian sections were stained with haematoxylin and eosin, according to the following protocol.

The tissue sections were dewaxed twice with xylol (10 min each passage) and hydrated with decreasing concentrations of ethanol (100%, 90%, and 70%, for 2 min each passage) and water (20 min). Hydrated sections were stained with Harris Haematoxylin (Thermo Scientific, Waltham, MA, USA, 72704) for 1 min, rinsed in tap water, and then stained with alcoholic eosin (Sigma-Aldrich, St. Louis, MO, USA, HT1101128-4L) at 1% for 45 s. Dehydration was performed with increasing concentrations of ethanol (70%, 90%, and 100%, for 10 s each passage) followed by two incubations with xylol (10 min each). Finally, the tissue sections were mounted in Entellan^®^ and air-dried. The sequential application of the dyes to the histologic sections resulted in the nuclei being stained blue, and cytoplasm and extracellular matrix in varying degrees of pink [23]. Haematoxylin and eosin staining was used for the ovarian morphology observation under a light microscope (Zeiss Axioskop 40, Jena, Germany) equipped with a digital camera (Zeiss AxioCam 208 Color). The representative images, at the ovarian midsection, were captured and used for the morphological evaluation and follicle counting at the ovarian midsection.

### 2.4. Follicle Counting at Ovarian Midsection

Haematoxylin and eosin slides were used for follicle counting. The follicles were branded as primordial, primary, secondary, or antral, and corpus luteum. The follicles were classified as primordial or primary when the oocytes were surrounded, respectively, by a single layer of squamous or cuboidal granulosa cells and secondary follicles when having more than one layer of granulosa cells with no visible antrum. Antral follicles were the ones that displayed areas of follicular fluid (antrum) or a single large antral space. *Corpora lutea* were identified as intraovarian bund structures with morphologically homogeneous round cells, showing an enhanced cytoplasm/nucleus ratio, when compared with granulosa cells, and deprived of oocyte. The number of primordial and primary follicles at the ovarian midsection was obtained by calculating the count of an ovarian midsection representative of each animal by two independent researchers.

### 2.5. Determination of Ovarian Midsection Area

The ovarian slides stained with haematoxylin and eosin were scanned and used to determine the ovarian midsection area. The ovarian area was quantified using the Image J software by the identification of the threshold cut point, with the blind intervention of the operator.

### 2.6. Sudan Black B Staining

The Sudan black B dye stained lipid-containing structures, giving them a dark black or brown colour. In paraffin-embedded tissues, the Sudan black B histochemical stain reacted against lipofuscin [24].

The ovarian sections were stained with Sudan black 0.1% for the lipofuscin examination. The tissue sections were dewaxed twice with xylol (10 min each passage) and hydrated with decreasing concentrations of ethanol (100%, 90%, and 70%, for 2 min each passage) and water (20 min). The sections were then stained for 50 min with 1% Sudan black B (Sigma-Aldrich, 199664) in 70% ethanol. Excess staining was removed by rinsing the slides in ethanol (70%) before a short incubation in phosphate-buffered saline (M–0.137 NaCl, 0.0027 KCl, 0.01 Na_2_HPO_4_, 0.0018 KH_2_PO_4_). The slices were mounted in 50% glycerol diluted in PBS and observed under a light microscope (Zeiss Axioskop 40) equipped with a digital camera (Zeiss AxioCam 208 Color). Representative images of the ovarian midsection were captured. Sudan black B staining was used to identify the ovarian cells that accumulate lipofuscin.

### 2.7. Autofluorescence Detection

The tissue sections were dewaxed twice with xylol (10 min each passage) and hydrated with decreasing concentrations of ethanol (100%, 90%, and 70%, for 2 min each passage) and water (20 min). Then, the slices were mounted in 50% glycerol diluted in phosphate-buffered saline and observed under a fluorescence microscope (Zeiss Axio Imager Z1) equipped with a digital camera (Zeiss AxioCam MRm). Autofluorescence was used to detect the lipofuscin deposition [25].

### 2.8. Statistical Analysis

Arithmetic means are given with a standard error of the mean (SEM). Statistical analyses were performed with GraphPad Prism 9.0.0 (GraphPad Software, San Diego, CA, USA). Unpaired Student’s *t*-test was used to compare between the groups. Sample correlations were evaluated by the Pearson rank correlation coefficient. The respective plots display a fitted linear regression line. A *p* < 0.05 was assumed to denote statistical significance.

## 3. Results

### 3.1. Morphological Analysis of Goats and Sheep Ovarian Tissue

We started the present study by evaluating the goats and sheep ovarian morphology in the juvenile and mature groups.

**Figure 1:**
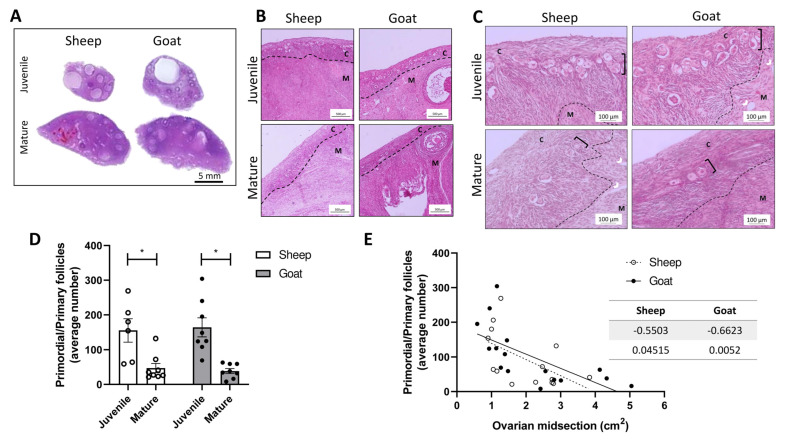
Sheep and goat ovarian structure and follicle pool. (**A**)—Representative ovarian midsections from juvenile and mature sheep and goats stained with haematoxylin and eosin. (**B**,**C**)—Representative ovarian midsections of juvenile and sheep and goats showing the distribution of primary and primordial follicles (right brackets) inserted in the ovarian cortex (letter C). Beneath the ovarian cortex, the ovarian medulla (letter M) with blood vessels is visible (arrowheads). Dash line separates the ovarian cortex from the medulla. (**D**)—Average number of primordial/primary follicles per animal and age group. A significant decrease in follicle pool was observed in mature sheep and goats (* *p* < 0.05). (**E**)—Negative correlation between the number of primordial/primary follicles and the area of ovarian midsection (*p* < 0.05).

The ovarian haematoxylin and eosin-stained midsections of juvenile and mature goats and sheep showed the presence of follicles at different stages of development (Figure 1A). The reason for the goat juvenile group having seven ovaries is due to the fact that we had to exclude one animal from the study because after the ovarian morphological examination, the absence of gonadotropin-dependent follicles was evident, and this denoted that the animal had not reached sexual maturity.

In sheep, primary and primordial follicles occupied predominantly the periphery and exhibited the usual characteristics. Primordial follicles showed a central small oocyte surrounded by flattened granulosa cells displaying single flattened nuclei; in primary follicles, beyond an increase in the oocyte size, granulosa cells exhibited a cuboid shape with a round central nucleus. In some follicles (secondary), additional granulosa cell layers were noticed and a few of them displayed emptied spaces, indicating that they were in the antral phase. The goat follicles had similar features. In both species, the central medulla had a heterogeneous stroma that included vessels, bundles of extracellular connective tissue, and many slender, fibroblast-like cells (Figure 1A–C). The average number of primary and primordial follicles count at the ovarian midsection is depicted in Figure 1D and the representative pictures are in Figure 1C. Mature animals displayed a significantly lower number of primordial and primary follicles in comparison with their juvenile peers.

As the precise age of each animal was unknown, we estimated the ovarian midsection and correlated it with the follicle reserve pool of the respective animal. A negative correlation between the size of the ovaries and the presence of primordial and primary follicles was observed (Figure 1E).

### 3.2. Identification of the Population of Cells Considered Hallmarks of Ovarian Ageing

**Figure 2:**
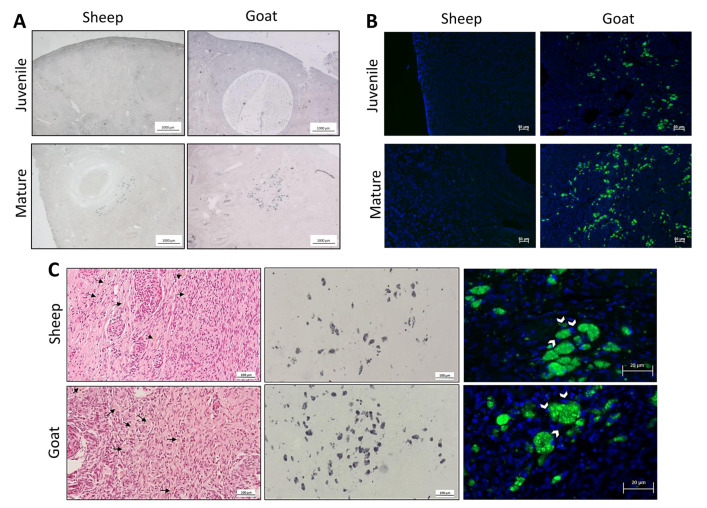
Identification of multinucleated giant cells in ovarian midsections. (**A**)—Representative images of ovary midsections of sheep and goats stained with Sudan black B. (**B**)—Representative images of ovarian autofluorescence. Ovarian autofluorescence is depicted in green and nuclear staining with DAPI in blue. (**C**)—Characterization of multinucleated giant cells. Multinucleated giant cells show a light dark brown cytoplasm when stained with haematoxylin and eosin (black arrows), lipofuscin accumulation (positivity for Sudan black B and autofluorescence emission) and are often multinucleated (white arrowheads).

Next, employing Sudan black B staining, a marker for the accumulation of lipofuscin in paraffin-embedded tissues, we were able to identify the presence of a unique population of stromal cells only in the ovaries of mature sheep and goats (Figure 2A). These cells appeared in clusters or elongated bundles and emitted auto-fluorescence when observed under the fluorescent microscope (Figure 2B). A closer observation, with higher amplifications, showed that these cells had a cytoplasm with a light-brown staining, a foamy appearance, and were often multinucleated (Figure 2C). Noteworthy was the absence of multinucleated giant cells in all the juvenile sheep and goats and their presence in the majority of the mature animals (Table 2).

## 4. Discussion

Biological ageing of the female reproductive system early in one’s lifetime is common among mammalian species, although the specific causes and consequences may vary [26,27,28,29]. Therefore, it is important to detect animal models which are physiologically closer to humans in order to improve the knowledge of reproductive ageing and to develop appropriate strategies aiming to delay it. The present study revealed that during sheep and goat reproductive ageing, the ovarian follicle pool is significantly reduced and the ovary undergoes morphological and molecular changes that are similar to what has been previously observed in the mice (the presence of a population of multinucleated giant cells considered to be the hallmarks of ovarian ageing). As important reproductive features of the goat and the sheep are physiologically closer to humans, this study reinforces the potential utility of these animal models to contribute to the improvement of the knowledge on reproductive ageing. Sheep, in particular, have been used in a variety of ovarian studies because the dimensions and structural organization of the ovaries [30,31], and the time required for folliculogenesis [32], evidence important similarities to humans.

Six-month-old juvenile animals were used in this study since in goats and sheep, puberty is reached between 4 and 12 months of age depending on several factors such as the breed, season of birth, and nutrition [33]. In this setting, while juvenile animals would be comparable to a young teenager, whereas 18- to 24-month-old animals, that still retain their reproductive potential [34], would correspond to an adult woman before the marked decrease in fertility that intensifies in the mid-30s [35]. Interestingly, the midsections of the ovaries of mature animals have a larger size when compared to juveniles; the difference parallels previous observations in humans, where ovarian morphometric studies show an earlier age-related increase in its volume, and a decrease afterwards, by 25–30 years of age [36,37]. In goat and sheep, to our knowledge, no additional data are currently available regarding ovarian morphometry and chronological age.

In parallel to humans, the morphological diversity of the follicles shows that they are at different functional stages and indicates that a continued attrition process is occurring, as is shown by the present study. In fact, in both species, at ages 18 to 24 months, i.e., much before the end of their reproductive potential [34], a significant reduction in the follicle reserve pool is already noted and results in a negative correlation between the ovarian follicle reserve pool and ovarian midsection area. Such a reduction in the follicle reserve pool during reproductive ageing is common to humans and mice [38,39] and is thus mainly due to continued primordial and primary follicle decrement and ovulations [39]. In contrast, much of the enlarged size of the ovarian midsection observed in older animals most likely results from the enlargement of the stroma, following fibroblast proliferation in response to the continuous process of ovarian tissue regeneration that occurs after ovulation.

Apart from the common connective tissue cells and fibers, multinucleated cells are an important component of the structural reorganization of the stroma. They have attracted considerable attention because they accumulate in the ovaries of mice and correlate negatively with the follicle pool [9,18,19]. These cells, considered as hallmarks of ovarian ageing [19], are here shown for the first time in the ovaries of mature sheep and goat. Interestingly, they are absent in the ovaries of juvenile animals. Additionally, important characteristics of such cells such as cell clustering, localization, lipofuscin accumulation (as revealed by Sudan black B positive staining and autofluorescence), and multiple nuclei were also confirmed in both species. The data obtained in this study indicate that they correspond to the multinucleated giant cells previously described in mice [9,18,19]. Although the characterization of multinucleated giant cells has not been extensive, they are positive for the F4/80 macrophage marker [18,21], which indicates that these multinuclear cells most likely result from the fusion of macrophages.

While ovarian macrophages play a role in tissue remodeling during follicle development, ovulation, and luteinization [40], macrophage fusion results from their inability to efficiently phagocytose their targets [41]. Therefore, it is possible that the progressive, age-related, ovarian malfunction underlies the appearance of those cells and their continued presence results in further unwanted effects. Indeed, macrophages secrete a variety of compounds that maintain a continued, low grade inflammatory and stressful environment; in addition, they also contribute to the removal of dead cells or their debris [42,43]. In any condition, this provides an opportunity for stromal cells and extracellular features to reorganize and enlarge, whose specific aspects would require additional studies.

Reproductive resemblances between humans and sheep or goat go much beyond our findings. Previous studies pointed out that the endocrine changes associated with ovarian ageing in sheep, as the increase in follicle stimulating the hormone levels and reduced secretion of ovarian inhibin A, are similar to those seen in peri-menopausal women [44]. Ageing was also associated with a follicle depletion together with a greater proportion of antral follicles, further emphasizing the usefulness of sheep and goats to study follicular population dynamics [44].

A major observation is that a goat ovary looks similar to a human ovary not only in size and tissue composition [45], but also in the aspects related to folliculogenesis [46,47]. The oocytes from the dominant follicles are released under the luteinizing hormone influence, while the subordinated follicles degenerate in a process of follicular atresia [48]. In addition, recent data indicate that the basic signalling pathway that leads to the activation of primordial follicles, the phosphatidylinositol 3-kinase/AKT serine-threonine kinase/mammalian target of the rapamycin (PI3K/AKT/mTOR) pathway activated by the granulosa cell-originated kit-ligand, is the same in women and sheep [49,50]. However, goats and sheep are polyoestrous females, showing a seasonal pattern in their reproductive activity. To minimise the contribution of seasonal patterns, the ovary collection was performed in the same period in both age groups (February–March), assuring that the animals were exposed to a similar photoperiod and only the average number of primordial and primary follicles (gonadotropin independent) in the representative midsection of the ovaries were counted.

Some of the advantages of using small ruminants as research models include easier housing and handling, lower maintenance expenses, and better acceptance in society when compared to larger animals, such as cows and horses [51]. Supporting these observations are the increasing number of publications using sheep and goats for testing bone implant material [52], the ovariectomised goat model for osteoporosis research [53,54], and the sheep model for the study of ovarian function preservation in the context of oncofertility. Employing cryoprotected stripes of ovary for controlled autotransplant studies, sheep ovaries have provided important ground information for the establishment and refinement of transplant techniques in the setting of the fertility preservation of women diagnosed with cancer and treated accordingly [32].

As previously mentioned, one drawback of working with sheep and goat to study reproductive ageing is the difficulty of obtaining samples from older animals, because their life span usually lasts while they are economically beneficial (reproductive capacity, milk production, and meat production), and not until they are at the end of their reproductive potential, which in sheep and goats averages 7 and 6 years, respectively [34]. Nevertheless, in the present work, the age-related comparison demonstrated the presence of multinucleated giant cells and a significant follicle pool reduction in animals aged between 18 and 24 months. As a whole, these observations parallel previous findings in mice [19] that show the presence of multinucleated giant cells before the decrement in reproductive capacity. Interest remains to characterize ovarian ageing at the end and after the loss of the reproductive span in the sheep and goat.

## 5. Conclusions

The present study confirmed the presence of multinucleated giant cells with lipofuscin accumulation and autofluorescent proprieties in the ovarian stroma of mature sheep and goat together with a reduction in the follicle reserve. These results should be considered whenever studies are carried out on the ovarian function in sheep and goats with a reproductive age higher than 1–2 years, as the presence of these hallmarks of ovarian ageing, adversely correlated to fertility in other species, can negatively contribute to the ovarian function.

## Figures and Tables

**Table 1:** Number of ovaries used in the current study, according to the specie and age.

	Sheep	Goat
Juvenile	8	7
Mature	8	8

**Table 2:** Percentage of ovaries positive for Sudan black B staining and autofluorescence.

	Sudan Black B		Autofluorescence	
	Sheep	Goat	Sheep	Goat
Juvenile	0%	0%	0%	0%
Mature	80%	67%	100%	62%

## Data Availability

The data used to support the findings of this study are available from the corresponding author upon request.
